# γ-tocotrienol inhibits angiogenesis-dependent growth of human hepatocellular carcinoma through abrogation of AKT/mTOR pathway in an orthotopic mouse model

**DOI:** 10.18632/oncotarget.1876

**Published:** 2014-03-31

**Authors:** Kodappully Sivaraman Siveen, Kwang Seok Ahn, Tina H. Ong, Muthu K. Shanmugam, Feng Li, Wei Ney Yap, Alan Prem Kumar, Chee Wai Fong, Vinay Tergaonkar, Kam M. Hui, Gautam Sethi

**Affiliations:** ^1^ Department of Pharmacology, Yong Loo Lin School of Medicine, National University of Singapore, Singapore; ^2^ College of Oriental Medicine, Kyung Hee University, Seoul 130-701, Republic of Korea; ^3^ Division of Cellular and Molecular Research, Humphrey Oei Institute of Cancer Research, National Cancer Centre, Singapore; ^4^ Cancer Science Institute of Singapore, Centre for Translational Medicine, 14 Medical Drive, #11-01M, Singapore; ^5^ School of Biomedical Sciences, Faculty of Health Sciences, Curtin University, Western Australia 6009, Australia; ^6^ Department of Biological Sciences, University of North Texas, Denton, Texas, 76203, USA; ^7^ Davos Life Science Pte Ltd, 3 Biopolis Drive; #04-19 Synapse, Singapore; ^8^ Institute of Molecular and Cell Biology, A*STAR, Biopolis Drive Proteos, Singapore; ^9^ Cancer and Stem Cell Biology Program, Duke–National University of Singapore Graduate Medical School, Singapore; ^10^ Department of Biochemistry, Yong Loo Lin School of Medicine, National University of Singapore, Singapore

**Keywords:** γ-tocotrienol, HCC, angiogenesis, AKT/mTOR, orthotopic model.

## Abstract

Angiogenesis is one of the key hallmarks of cancer. In this study, we investigated whether γ-tocotrienol can abrogate angiogenesis-mediated tumor growth in hepatocellular carcinoma (HCC) and if so, through what molecular mechanisms. We observed that γ-tocotrienol inhibited vascular endothelial growth factor (VEGF)-induced migration, invasion, tube formation and viability of HUVECs *in vitro*. Moreover, γ-tocotrienol reduced the number of capillary sprouts from matrigel embedded rat thoracic aortic ring in a dose-dependent manner. Also, in chick chorioallantoic membrane assay, γ-tocotrienol significantly reduced the blood vessels formation. We further noticed that γ-tocotrienol blocked angiogenesis in an *in vivo* matrigel plug assay. Furthermore, γ-tocotrienol inhibited VEGF-induced autophosphorylation of VEGFR2 in HUVECs and also suppressed the constitutive activation of AKT/mammalian target of rapamycin (mTOR) signal transduction cascades in HUVECs as well as in HCC cells. Interestingly, γ-tocotrienol was also found to significantly reduce the tumor growth in an orthotopic HCC mouse model and inhibit tumor-induced angiogenesis in HCC patient xenografts through the suppression of various biomarkers of proliferation and angiogenesis. Taken together, our findings strongly suggest that γ-tocotrienol might be a promising anti-angiogenic drug with significant antitumor activity in HCC.

## INTRODUCTION

Angiogenesis, the process of formation of new blood vessels from preexisting vessels, is an essential event in a variety of physiological processes like embryonic development, ovulation and wound healing, as well as pathologic conditions such as cancer, chronic inflammation, arthritis, aneurysms and arteriovenous malformations [1, 2]. It is now well established that angiogenesis is vital for invasive tumor growth and metastasis and contributes significantly to cancer progression [3, 4]. For tumors to develop in size and achieve metastatic potential they must make an “angiogenic switch”, a complex cascade of events, which is turned on when the concentration of natural pro-angiogenic factors outweighs that of anti-angiogenic [3, 5]. The newly generated blood vessels supply adequate oxygen and nutrition, supporting the growth of tumor mass, and later aid in the initiation of metastasis, which contributes to >90% of deaths in various cancers including hepatocellular carcinoma (HCC) [6, 7]. Although numerous pro-angiogenic factors have been identified as potential mediators of the angiogenic switch, the vascular endothelial growth factor (VEGF) appears to be a key factor in pathological situations that involve tumor neovascularization [8]. VEGF is a specific mitogen and survival factor for endothelial cells *in vitro*, capable of directly stimulating their growth and also acts as a potent angiogenic factor while promoting angiogenesis *in vivo* [6, 9]. VEGF regulates angiogenesis mainly via two interacting tyrosine kinase receptors, vascular endothelial growth factor receptor 1 (VEGFR1) and vascular endothelial growth factor 2 (VEGFR2), but its signal transduction and biological responses are mediated primarily via VEGFR2 [9, 10].

HCC's are highly vascular tumors with high microvessel density and levels of circulating VEGF, thus making the angiogenesis pathway an attractive therapeutic target [11, 12]. A number of angiogenesis blockers, including small molecule kinase inhibitors and monoclonal antibodies are currently being evaluated as potential therapeutic agents against HCC [11, 13-15]. Treatment with endogenous angiogenic inhibitors such as endostatin and angiostatin has been found to reverse the angiogenic switch thereby significantly preventing growth of tumor vasculature [15, 16]. However, most of the anti-angiogenic therapies currently available for treatment have significant side effects [14, 17]. So the identication of pharmacological agents targeting angiogenesis is considered an important strategy both for cancer prevention and treatment.

Small molecules derived from natural products provide a potential drug pool in the development of new bioactive molecules [18]. In the present study, we investigated the anti-invasive, anti-angiogenic and anticancer potential of a vitamin E derivative, γ-tocotrienol derived from palm oil in endothelial and HCC cell lines and orthotopic mouse model. Increasing evidences indicate that γ-tocotrienol exerts significant antiprolifeartive/pro-apoptotic effects in diverse cancers, including breast, liver, lung, gastric, colorectal, skin and prostate cancers [19-22] through the negative regulation of various oncogenic molecules including NF-κB [23, 24], STAT3 [25], telomerase [26], peroxisome proliferator-activated receptor gamma [27], hypoxia inducible factor-1alpha [28], Wnt/β-catenin [29], epidermal growth factor [22] and inhibitor of differentiation family proteins [30]. Although, few prior studies have indicated that palm tocotrienols can inhibit angiogenesis [31-33] and decrease levels of pro-angiogenic markers [34], but the underlying molecular mechanisms and whether γ-tocotrienol specifically affects tumor angiogenesis and growth in HCC has never been studied before. We observed that γ-tocotrienol can indeed attenuate endothelial cell proliferation, migration, invasion and tube formation through the abrogation of VEGFR2-mediated AKT/mTOR signaling cascades. *In vivo*, γ-tocotrienol inhibited chick chorioallantoic membrane (CAM) and VEGF-induced angiogenesis and the growth of tumor in an orthotopic HCC mouse model through the modulation of various oncogenic biomarkers.

## RESULTS

### γ-tocotrienol suppresses VEGF-induced migratory and invasive potential of HUVEC *in vitro*

**Figure 1:**
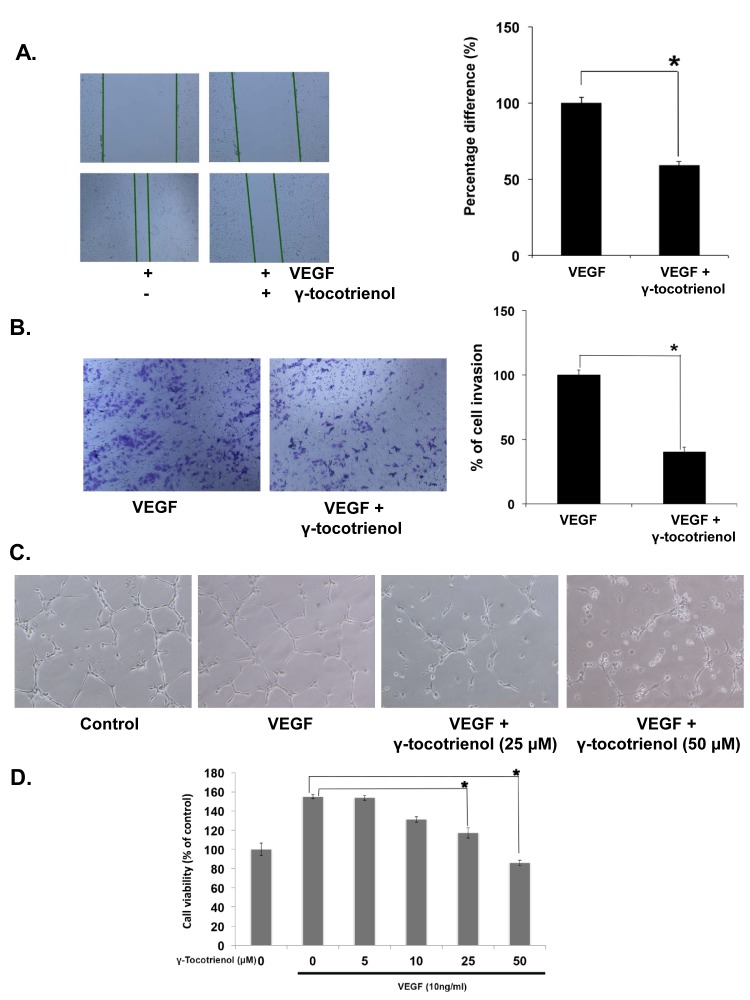
γ-tocotrienol inhibits VEGF-induced endothelial cell migration, invasion, capillary structure formation *in vitro* and cell viability A, γ-tocotrienol inhibited HUVEC migration. An IBIDI culture insert (IBIDI GmbH) consists of two reservoirs separated by a 500 μm thick wall created by a culture insert in a 35mm petri dish. An equal number of HUVECs (70 μl; 5×10^5^ cells/ml) were added into the two reservoirs of the same insert and incubated at 37°C/5% CO2. After 12 hours, the insert was gently removed creating a gap of ~500 μm. The cells were treated with 50 μM γ-tocotrienol for 12 h before being exposed to 10ng/mL VEGF for 24 h. Width of wound was measured at time zero and 24h of incubation with and without γ-tocotrienol. The representative photographs showed the same area at time zero and after 24 h of incubation. B, γ-tocotrienol inhibited HUVEC invasion through matrigel coated polycarbonate membrane. After pre-incubation with or without 50 μM γ-tocotrienol for 12 h, transwell chambers were then placed into the wells of a 24-well plate, in which we had added Medium 200 containing 10 ng/mL VEGF. After incubation for 24h cell invasion was analyzed and columns represent mean number of invaded cells. C, γ-Tocotrienol inhibited the VEGF-induced tube formation of endothelial cells in matrigel. After incubation, endothelial cells were fixed, and tubular structures were photographed (magnification, ×100). D, γ-Tocotrienol significantly inhibited the VEGF-induced cell survival of HUVECs. Cell viability was determined by MTT assay. *, p < 0.01 versus VEGF alone.

Endothelial cell migration is one of the most important and early event during the process of angiogenesis [8]. To assess the *in vitro* anti-angiogenic property of γ-tocotrienol, we examined its effects on the chemotactic motility of endothelial cells using the wound-healing migration and invasion assays. When HUVEC migration was stimulated with VEGF, the wound closing in cells treated with γ-tocotrienol was much less when compared to control (VEGF alone) (Fig. 1A). Similar results were obtained when HUVECs treated with γ-tocotrienol were allowed to invade the matrigel coated polycarbonate membrane (Fig. 1B).

### γ-tocotrienol abrogates VEGF-induced HUVEC capillary-like structure formation and viability *in vitro*

HUVECs can also spontaneously form three dimensional capillary-like tubular structures when cultured on matrigel. Tube formation assay represents a simple, reliable and powerful model for studying inhibitors of angiogenesis and so we studied the effects of γ-tocotrienol on tubulogenesis in HUVECs. Our results indicated that HUVECs can form robust tubular-like structures when seeded on growth factor–reduced two-dimensional matrigel, in the presence of VEGF. However, treatment with γ-tocotrienol resulted in significant reduction in the number and the continuity of HUVEC capillary-like structures in a dose-dependent manner (Fig. 1C), suggesting that *in vitro* HUVEC tube formation is inhibited. The process of angiogenesis also requires the proliferation of endothelial cells, so we examined the effect of γ-tocotrienol on VEGF-induced proliferation of HUVEC cells. As shown in Fig. 1D, treatment of HUVECs with γ-tocotrienol resulted in a dose-depend reduction in VEGF-induced cell viability. Overall, these findings clearly demonstrated that γ-tocotrienol can cause significant inhibition of VEGF-induced migration, invasion, tube formation and proliferation of HUVECs.

### γ-tocotrienol inhibits VEGF-induced microvessel formation *ex vivo*

**Figure 2:**
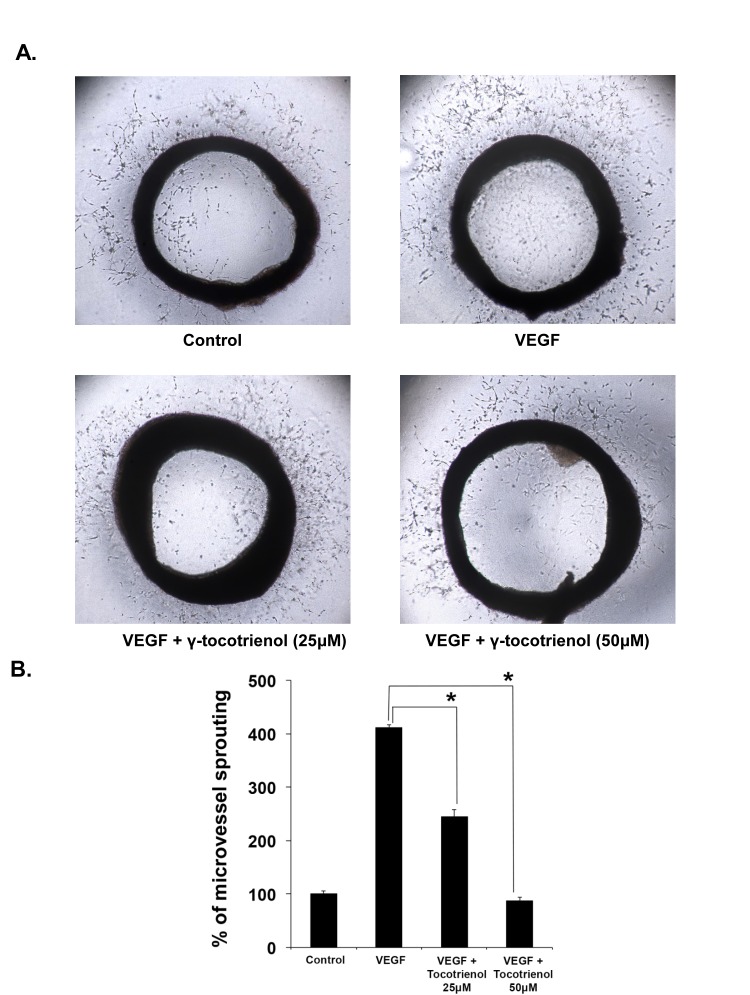
γ-Tocotrienol inhibits VEGF-induced microvessel sprouting *ex vivo* Aortic segments isolated from Sprague-Dawley rats were placed in the Matrigel coated plated and then overlayed with matrigel and treated with VEGF in the presence or absence of γ-tocotrienol. A, Representative photographs of sprouts from the margins of aortic rings. B, The number of sprouts were counted manually and epressed as a bar diagram. *, p < 0.01 versus VEGF alone.

**Figure 3:**
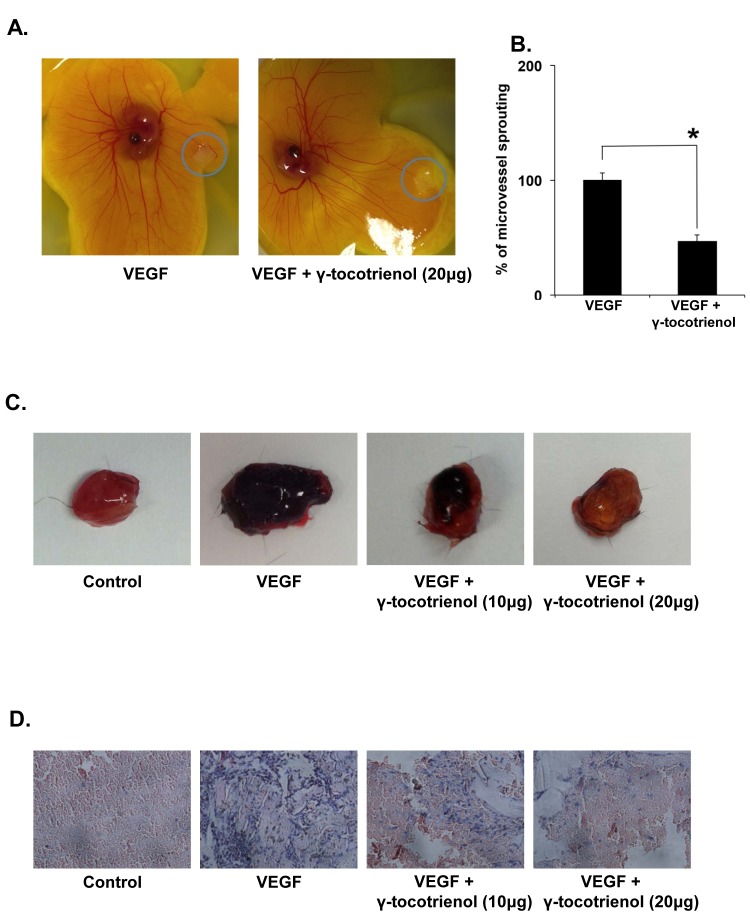
A, γ-tocotrienol inhibits angiogenesis in the chick chorioallantoic membrane (CAM) assay The rectangular filters papers contained VEGF alone or in combinaiton with γ-Tocotrienol. The representative photographs of control and γ-tocotrienol-treated CAMs were shown. B, The number of the microvessels was quantified manually and displayed as bar diagram *p < 0.01 versus VEGF alone. C, γ-Tocotrienol inhibits VEGF-induced angiogenesis *in vivo*. Six-week-old C57/BL/6 mice were injected with 0.5 mL of matrigel containing 10 or 20 μg γ-tocotrienol, 100 ng of VEGF, and 20 units of heparin into the ventral area (n = 5 per group). After 6 days, the skin of mice was pulled back to expose the intact Matrigel plugs and were photographed. D, γ-Tocotrienol inhibited blood vessel formation. The Matrigel plugs were fixed, sectioned, and stained with H&E (magnification, ×200).

We further explored the anti-angiogenic activity of γ-tocotrienol using 2 *ex vivo* angiogenesis models, the rat thoracic aortic ring and the chick embryo chorioallantoic membrane assays. The serum-free three-dimensional rat aortic model closely approximates the complexities of angiogenesis *in vivo*, from endothelial activation to pericyte acquisition and remodelling. So we analyzed the sprouting of micro-vessels from aortic rings in the presence or absence of γ-tocotrienol. VEGF significantly stimulated microvessel sprouting, leading to the formation of a network of vessels around the aortic rings (Fig. 2A). The presence of γ-tocotrienol at different concentrations significantly antagonized the VEGF-induced sprouting in a dose-dependent manner as evidenced by a decrease in the numbers of microvessel spouting as shown in Fig. 2B. In the chorioallantoic membrane (CAM) assay, the fertilized chicken eggs can form blood vessels, especially microvessels and serve as an ideal indicator of the anti- or pro-angiogenic properties of drugs. As shown in Fig. 3A and 3B, treatment with γ-tocotrienol significantly reduced the number of microvessels when compared to VEGF alone control, demonstrating that γ-tocotrienol can indeed inhibit CAM induced angiogenesis.

### γ-tocotrienol attenuates VEGF-induced angiogenesis *in vivo*

The effect of γ-tocotrienol on VEGF-induced angiogenesis *in vivo* was also studied using matrigel plug assay. As shown in Fig. 3C, matrigel plugs containing VEGF alone appeared dark red, indicating that functional vasculatures had formed inside the matrigel via angiogenesis triggered by VEGF. In contrast, the addition of different concentrations of γ-tocotrienol (10 or 20 μg per plug) to the matrigel plugs containing VEGF dramatically inhibited vascular formation. These plugs displayed much paler appearance at 10 μg and showed almost no vascular formation at 20 μg. H&E staining of the functional vasculature in Matrigel plugs (Fig. 3D) showed that γ-tocotrienol at a dose of 20 μg dramatically blocked VEGF-induced vasculature formation *in vivo*.

### γ-tocotrienol inhibits the activation of AKT/mTOR signaling cascade in endothelial cells

**Figure 4:**
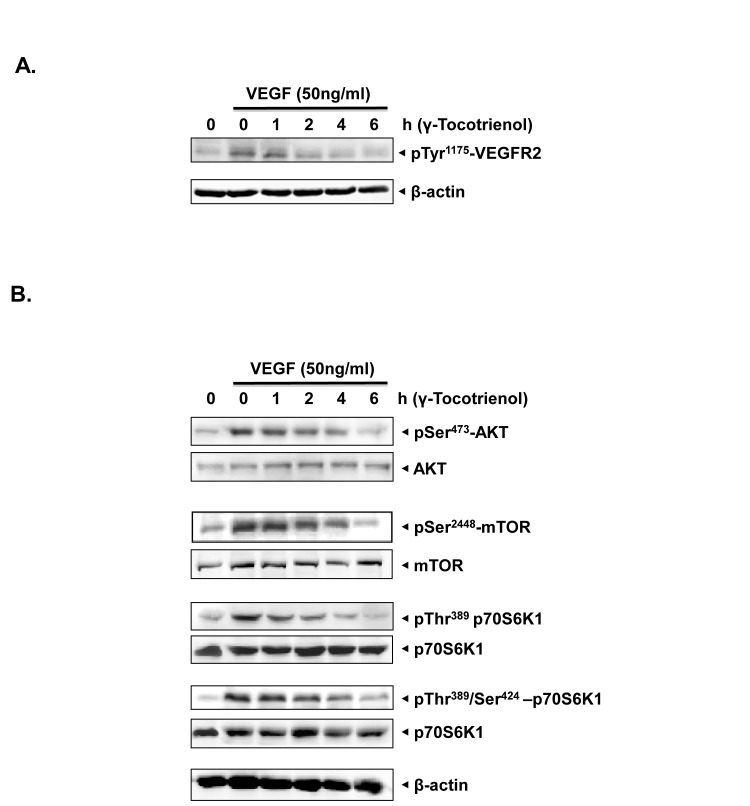
A, γ-tocotrienol suppressed the activation of VEGFR2 induced by VEGF in a time dependent manner The activation status of VEGFR2 was tested by western blot analysis and probed with anti–phosphorylated VEGFR2 antibody. The same blots were stripped and reprobed with β-actin antibody to verify equal protein loading. B, γ-tocotrienol inhibited the activation of AKT/mTOR signaling pathway in endothelial cells. Proteins from different treatments were probed with phospho-specific antibodies. The same blots were stripped and reprobed with antibody against total protein to verify equal protein loading. The results shown are representative of two independent experiments.

To elucidate the molecular basis of anti-angiogenic activity, we examined the signaling cascades modulated by γ-tocotrienol in HUVECs using western blot analysis. In tumor microenvironment, one of the most potent mediators of the angiogenic switch is VEGF, which mediates its signaling events through VEGFR2 phosphorylation [9]. VEGFR-2 is thought to be the primary receptor involved in angiogenesis, and its activation is considered critical in endothelial cell migration, proliferation and survival [10], hence we next examined the effect of γ-tocotrienol on VEGFR2 activation. The phosphorylation of VEGFR2 at Try1175 residue was suppressed by γ-tocotrienol in a time-dependent manner (Fig. 4A). These results indicated that the observed anti-angiogenic effects of γ-tocotrienol may be partially mediated through inhibition of VEGR2 activation.

Our next series of experiments were aimed at investigating the effects of γ-tocotrienol on the key components of the downstream signaling pathway that regulate the endothelial cell function in angiogenesis. We found that γ-tocotrienol effectively suppressed VEGF-triggered activation of AKT/mTOR signaling cascade, including AKT, mTOR, and P70S6 kinases in HUVECs in a time-dependent manner (Fig. 4B), suggesting that anti-angiogenic effects of γ-tocotrienol may be mediated through negative regulation of the AKT/mTOR signaling cascade.

### γ-tocotrienol induces apoptosis and inhibits AKT/mTOR signaling pathway in HCC cells

**Figure 5:**
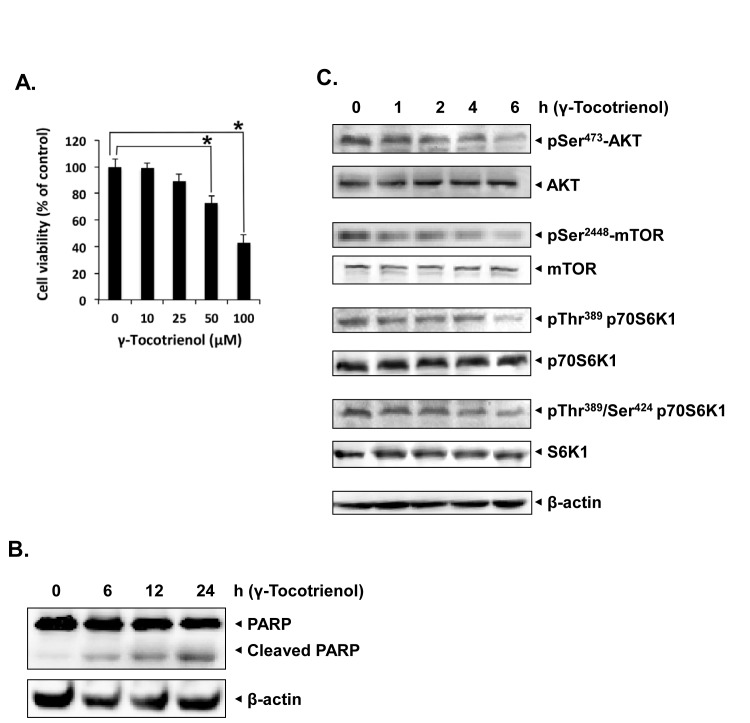
γ-tocotrienol induces apoptosis and inhibits AKT/mTOR pathway in HCC cells A, γ-tocotrienol inhibited cell viability of HCCLM3 cells in a dose dependent manner. Cell viability was quantified by MTT assay. Columns, mean from three different experiments; bars, SD. B, γ-tocotrienol induces apoptosis in HCCLM3 cells in a time-dependent as evident by western blot analysis of PARP. HCCLM3 cells were treated with 50 μM γ-tocotrienol for the indicated times, and whole-cell extracts were prepared, separated on SDS-PAGE, and subjected to western blot analysis against PARP antibody. The same blot was stripped and reprobed with β-actin antibody to show equal protein loading. C, γ-tocotrienol suppressed the phosphorylation of mTOR signaling pathway kinases in HCCLM3 cell. HCCLM3 cells were treated with 50 μM γ-tocotrienol for the indicated times, and whole-cell extracts were prepared, separated on SDS-PAGE, and subjected to western blot analysis against phosphor-specific antibodies for AKT, mTOR and P70S6K1. The same blots were stripped and reprobed with antibodies for AKT, mTOR and P70S6K1 to show equal protein loading. The results shown are representative of two independent experiments.

Whether γ-tocotrienol can induce apoptosis through the abrogation of AKT/mTOR pathway in HCC cells was investigated next. MTT analysis showed that γ-tocotrienol caused a significant dose-dependent inhibition in the proliferation of highly metastatic HCCLM3 cells (Fig. 5A). We also noticed that γ-tocotrienol significantly induced apoptosis in HCCLM3 cells as evident by substantial cleavage of full-length PARP (116 kDa) into its large cleavage fragment (89 kDa) (Fig. 5B). These findings suggest that γ-tocotrienol also has direct apoptotic effects on HCC cells besides its anti-angiogenic effect on endothelial cells. Treatment with γ-tocotrienol also inhibited the activation of AKT, mTOR, and P70S6K kinases in a time-dependent manner in HCCLM3 cells (Fig. 5C), suggesting that the AKT/mTOR pathway may mediate both anti-angiogenic/anti-tumor effects of γ-tocotrienol.

### γ-tocotrienol suppresses tumor growth in an orthotopic HCC mouse model

**Figure 6:**
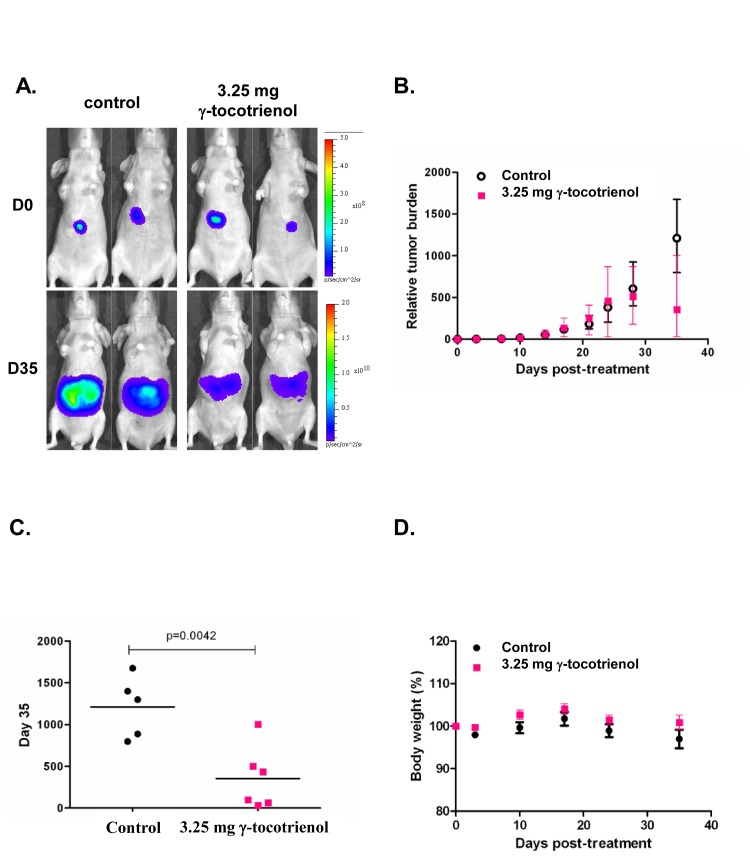
*In vivo* antitumor activities of γ-tocotrienol in mice orthotopically implanted with the human HCCLM3 tumor A) Representative bioluminescent images of control and treated mice. D= day. The colour scale depicts the photon flux (p/s) emitted. B) Tumor growth in Balb/c nude mice bearing orthotopically implanted HCCLM3-Luc tumors treated with vehicle alone (n = 5) or 3.25 mg γ-tocotrienol (n = 6). Point= mean; bars= SE. C) Relative tumor burden at time of euthanasia. Bar= mean. D) Observed body weight (baseline set as 100%). Values are given as means ± SEMs for each group.

We tested the antitumor potential of γ-tocotrienol *in vivo* in an orthotopic model with the human HCCLM3 cells that stably expressed the firefly luciferase gene to enable the non-invasive monitoring of tumor growth. Prior to the first therapeutic injection (10 days after tumor implantation), the presence of growing orthotopic tumors could be detected and are mainly confined to the liver (Fig. 6A). Mice were treated five times a week with either vehicles alone (n = 5) or 3.25 mg (n = 6) of γ-tocotrienol for up to five weeks. Bioluminescence imaging revealed the significant inhibition of tumor growth in the γ-tocotrienol-treated group compared with the vehicle-treated control group (Fig. 6A). Quantitative analysis at set time points were quantitated by measuring photon counts and expressed as tumor burden relative to photon counts before the first therapeutic injection (Fig. 6B). It was determined that γ-tocotrienol at 3.25 mg induced significant inhibition of tumor growth compared with the vehicle-treated controls, p=0.0042 (Fig. 6C). No significant changes in body weight were observed in the treated mice, showing that γ-tocotrienol produced insignificant toxicity in the treated mice at the curative dose (Fig. 6D).

### γ-tocotrienol abrogates the expression of biomarkers of proliferation and angiogenesis in tumor tissues

**Figure 7:**
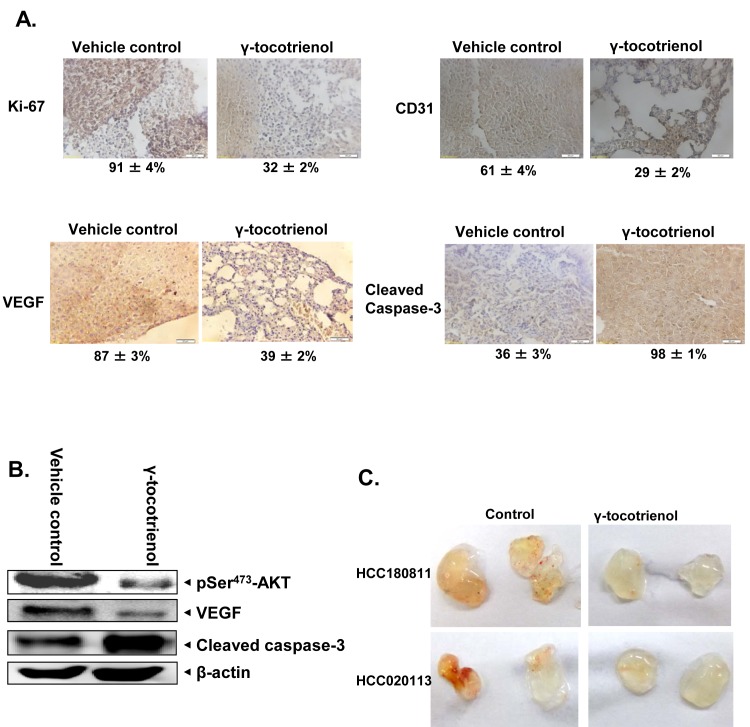
γ-tocotrienol modulates the expression of various oncogenic biomarkers in tumor tissues A) Immunohistochemical analysis of Ki-67, VEGF, CD31, and cleaved caspase-3 showed the inhibition in expression of Ki-67, VEGF, and CD31 and increased levels of cleaved caspase-3 expression in γ-tocotrienol treated samples as compared with control group. Percentage indicates positive staining for the given biomarker. The photographs were taken at the magnification of 40 X. B) Western blot analysis of p-AKT, VEGF and cleaved caspase-3 proteins indicated a decrease in the expression of p-AKT and VEGF and increase in levels of cleaved caspase-3 expression in γ-tocotrienol treated samples as compared with control group. C) Effect of γ-tocotrienol on tumor-induced angiogenesis *in-vivo*. SCID mice were injected with 0.4 ml of Matrigel containing 5 × 10^6^ single cell suspensions freshly derived from HCC patients (designated as HCC180811 and HCC 020113) and 20 units heparin with or without γ-tocotrienol (20 μg). Gross appearance of matrigel plugs retrieved from SCID mice 7 days post-injection were shown.

We next evaluated the effect of γ-tocotrienol on the expression of Ki-67 (marker of proliferation), VEGF, CD31 (markers of angiogenesis) and caspase-3 (marker of apoptosis) in HCC tumor tissues by immunohistochemical analysis. As shown in Fig. 7A, expression of Ki-67, VEGF, and CD31 was downregulated and that of cleaved caspase-3 was significantly increased in γ-tocotrienol treated group as compared with control group. We also analyzed the effect of γ-tocotrienol on constitutive p-AKT levels in HCC tumor tissues by western blot analysis and found that γ-tocotrienol substantially inhibited the constitutive AKT activation in treated group as compared with control group (Fig. 7B). Also treatment with γ-tocotrienol led to a decrease in the expression of VEGF protein and increase in the expression of cleaved casapse-3 which further substantiates it potential as an anti-angiogenic and pro-apototic agent in HCC.

### γ-tocotrienol inhibits tumor-induced angiogenesis in SCID mice

To evaluate the ability of γ-tocotrienol to inhibit tumor-induced angiogenesis, a xenograft tumor-induced angiogenesis model was employed. Single cell suspensions were prepared from freshly derived HCC patients' xenografts, tentatively designated as HCC180811 and HCC020113. The HCC cells prepared were mixed with matrigel and injected into the flanks of mice with or without γ-tocotrienol. Gel plugs were harvested 7 days after implantation. Both cell suspensions induced blood vessel formation in the plug (Fig. 7C). However, blood vessel formations induced by the cell suspensions were significantly reduced in the presence of 20 ug γ-tocotrienol (Fig. 7C).

## DISCUSSION

The goal of this study was to examine whether γ-tocotrienol can inhibit the angiogenesis mediated growth of HCC carcinoma through abrogation of AKT/mTOR pathway in an orthotopic mouse model. Our results suggest that γ-tocotrienol is a potent angiogenesis inhibitor and inhibits multiple steps of angiogenesis, including endothelial cell viability, migration, invasion, and differentiation into capillary like structures. γ-tocotrienol was found to exert its anti-angiogenic effects via targeting AKT/mTOR signaling cascade in endothelial HCC cells. We also observed for the first time that γ-tocotrienol can significantly suppress tumor growth in an orthotopic human HCC mouse model through the suppression of various mediators of proliferation, angiogenesis and survival.

The process of angiogenesis has been recognized to play a crucial role in cancer progression as the newly formed tumor vasculature serve initially as feeding tubes providing nutrients and oxygen supply for the growing tumor mass and finally as conduits for dissemination of tumor cells that have escaped from the established primary tumor [17]. The current strategies for anticancer therapy become ineffective once tumor cells reach favored secondary organs and generate metastatic foci. Therefore control of tumor angiogenesis has become a central issue in the fight against cancer progression [14]. Anti-cancer drugs such as bevacizumab (anti-VEGF monoclonal antibody) and vatalanib (VEGFR inhibitor) have already been successfully approved for clinically treating many malignant tumors by targeting anti-angiogenic mediators [15, 16]. However, the identification of novel anti-angiogenic agents with anti-tumor activity and less side effects is urgently needed.

Endothelial cells, which are the major components making up blood vessels, proliferate rapidly during angiogenesis than under normal conditions. As VEGF is the major mediator of tumor associated angiogenesis, we analyzed the rate of proliferation and viability of HUVECs stimulated with VEGF. The ability of γ-tocotrienol to reduce the VEGF-induced proliferation of endothelial cells indicated the ability of this vitamin E isoform to abrogate the formation of tumor vasculature. Treatment of HUVECs with γ-tocotrienol effectively abrogated VEGF-induced migration, invasion, and capillary-like structures formation *in vitro*. VEGF generally exerts its biological effects by binding to transmembrane receptors such as VEGFR1 and VEGFR2, both of which are specifically expressed on the surfaces of endothelial cells and contain a cytoplasmic tyrosine kinase domain [15]. The binding of VEGF to VEGFRs leads to conformational changes in the receptors, followed by dimerization and autophosphorylation of the tyrosine residues [8]. Interestingly, we found that γ-tocotrienol substantially down-regulated the VEGF-induced phosphorylation of VEGFR2 in HUVECs, thereby indicating that the anti-angiogenic effects of this Vitamin E isoform may be partially mediated through inhibition of VEGR2 activation. We also further validated the anti-angiogenic properties of γ-tocotrienol using *ex vivo* rat thoracic aortic ring capillary formation, CAM, and the *in vivo* matrigel plug assays.

The PI3K/AKT/mTOR pathway is involved in the regulation of multiple cellular processes, including cell proliferation, migration, invasion and survival. In many cancers this pathway is overactive, reducing apoptosis, allowing proliferation and thus enhanced signaling through this pathway is a significant contributor to new blood vessel formation [35, 36]. The activation of the AKT/mTOR/p70S6 kinase pathway during normal blood vessel development can promote angiogenesis by increasing VEGF expression in a HIF-1α dependent and independent manner [37]. A previous study reported that mTOR, an important downstream target of PI3K/AKT, can also increase HIF-1α-dependent gene expression in certain tumor cell types [38]. Mammalian mTOR is known to regulate translation through p70S6K1 and the eukaryotic translation initiation factor 4E-binding proteins. P70S6K1 activation is found in various human cancers such as thyroid cancer, breast cancer, and ovarian cancer and P70S6K1 plays an important role in phosphatase and tensin homologue (PTEN)-negative and in AKT-overexpressing tumors [36, 39]. Recent studies also suggest that P70S6 kinase, downstream of mTOR, drives the expression of HIF-1α and VEGF in human ovarian cancer, mediating tumor growth and angiogenesis. We found that treatment with γ-tocotrienol substantially reduced the activation of AKT/mTOR/ P70S6 kinases in endothelial cells in a time-dependent manner. Although, prior studies have indicated that palm tocotrienols can inhibit angiogenesis [31-33, 40] and decrease levels of pro-angiogenic markers [28, 34], the exact molecular mechanisms of these anti-angiogenic effects have never been investigated before. Our findings that γ-tocotrienol can significantly reduce VEGF-induced migration, invasion, and angiogenesis through the modulation of AKT/mTOR pathway is contrary to a previously published study in which this Vitamin E isoform was shown to exert its anti-angiogenic effects via down-regulation of β-catenin pathway [33], whereas δ-tocotrienol isoform acted similarly by reducing HIF-1α-protein expression or increasing HIF-1α-degradation [28].

We further noticed that γ-tocotrienol treatment also induced substantial apoptosis in HCCLM3 cells, which are fairly resistant to conventional chemotherapeutic agents as evident by PARP cleavage assay. These results are consistent with our previous report in which γ-tocotrienol was found to induce apoptosis in another HCC cell line, namely HepG2 [25]. We also report for the first time that γ-tocotrienol treatment at a dose of 3.25 mg per mice (equivalent to around 700 mg dose in a 60 kg human) significantly suppressed HCC growth in orthotopic mouse model and downregulated the expression of various biomarkers of proliferation and angiogenesis in tumor tissues. Western blot analysis data also revealed that γ-tocotrienol treatment can substantially inhibit the expression of phospho-AKT and VEGF, and increase the expression of cleaved caspase-3 in tumor tissues as compared to the control. We also demonstrate for the first time the potential of γ-tocotrienol to inhibit tumor-induced angiogenesis in HCC patient xenografts implanted in SCID mice.

To the best of our knowledge, no prior studies with γ-tocotrienol have been conducted in HCC mouse models, and our observations clearly indicate that γ-tocotrienol holds enormous promise for the treatment of HCC through the abrogation of AKT/mTOR pathway. These observations are consistent with prior reports in which γ-tocotrienol has been found to be well tolerated in pre-clinical studies, with no reported toxicity [19, 41, 42].

## CONCLUSIONS

Thus, overall, our experimental findings clearly indicate that the anti-cancer effects of γ-tocotrienol in HCC are mediated through the mitigation of AKT/mTOR signaling cascade and thus provide a strong rationale for pursuing the use of γ-tocotrienol in the treatment of HCC and other malignancies where angiogenesis is the key contributor to disease progression. Because γ-tocotrienol is non-toxic and currently undergoing clinical trials, the present study would provide the basis of novel therapeutic options for the treatment of HCC patients.

## MATERIALS AND METHODS

### Reagents

γ-tocotrienol with purity more than 97% was obtained from Davos Life Science, Singapore in the form of Naturale^3^ γ-tocotrienol. γ-tocotrienol was dissolved in dimethylsulfoxide (DMSO) as a 10 mM stock solution and stored at 4°C for the *in vitro* experiments. Further dilution was done in cell culture medium as required. Human recombinant vascular endothelial growth factor (VEGF) and growth factor reduced (GFR) matrigel matrix were purchased from BD Biosciences (CA, USA). Mayer's hematoxylin solution, eosin Y Solution, MTT, Tris, glycine, NaCl, SDS, BSA, and β-actin antibody were purchased from Sigma-Aldrich (St. Louis, MO). Medium 200, low serum growth supplement (LSGS), DMEM medium, fetal bovine serum (FBS), 0.4% trypan blue vital stain, and antibiotic-antimycotic mixture were obtained from Invitrogen (Carlsbad, CA). Rabbit polyclonal antibodies to phospho-AKT (Ser 473), phospho-mTOR (Ser 2448), phospho-p70 S6 Kinase (Thr 389), phospho-p70 S6 Kinase (Thr421/Ser424), AKT, mTOR, p70S6K1 and CD31 were purchased from Cell Signaling Technology (Beverly, MA). Antibodies against VEGF, Ki-67, PARP, cleaved caspase-3, goat anti-rabbit-horse radish peroxidase (HRP) conjugate, and goat anti-mouse HRP were purchased from Santa Cruz Biotechnology (San Diego, CA).

### Cell lines

Primary human umbilical vascular endothelial cells (HUVECs) were obtained from Invitrogen (Carlsbad, CA). HUVECs were cultured in Medium 200 supplemented with LSGS. HCCLM3 cell lines were a kind gift from Professor Zhao-You Tang at the Liver Cancer Institute (Zhongshan Hospital, Fudan University, Shanghai) and have been described previously[43]. HCCLM3 were cultured in high glucose DMEM containing 1× antibiotic-antimycotic solution with 10% FBS. HUVECs and HCCLM3 cells were cultured at 37°C under a humidified 95%:5% (v/v) mixture of air and CO_2_.

### Wound healing migration assay

The migration of cells was investigated using a wound healing assay. An IBIDI culture insert (ibidi GmbH, Munich, Germany) consists of two reservoirs separated by a 500 μm thick wall created by a culture insert in a 35 mm petri dish. For migration assay, HUVECs were starved to inactivate cell proliferation and then an equal number of cells (70 μl; 5×10^5^ cells/ml) were added into the two reservoirs of the same insert and incubated at 37°C/5% CO_2_. After 12 h, the insert was gently removed creating a gap of ~500 μm. The cells were treated with γ-tocotrienol (50 μM) for 12 h before being exposed to VEGF (10 ng/mL) for 24 h. After incubation, the wounds were observed using bright field microscopy and multiple images were taken at areas flanking the intersections of the wound. Gap distance of the wound was measured at three different sites using Photoshop software, and the data were normalized to the average of the control. Graphs were plotted against the percentage of migration distance the cells moved before and after treatment, normalized to control.

### Invasion assay

The *in vitro* invasion assay was performed using Bio-Coat matrigel invasion assay system (BD Biosciences, San Jose, CA, USA), according to the manufacturer's instructions. 5×10^4^ HUVECs were suspended in Medium 200 and seeded into the Matrigel transwell chambers consisting of polycarbonate membranes with 8 μm pores. After pre-incubation with or without γ-tocotrienol for 12 h, the transwell chambers were then placed into appropriate wells of a 24 well plate, in which either the basal medium only or basal medium containing 10 ng/mL VEGF had been added. After incubation, the upper surfaces of the Transwell chambers were wiped with cotton swabs, and the invading cells were fixed and stained with crystal violet solution. The invading cells were then counted in five randomly selected areas under microscopic observation as described previously [44].

### Capillary-like tube formation assay

Tube formation was assessed as described previously [45]. Briefly, HUVECs were pretreated with various dilutions of γ-tocotrienol for 12 h and then seeded onto the Matrigel layer in 24-well plates at a density of 5 × 10^4^ cells in Medium 200 with or without VEGF. After 6 h, tubular structure of endothelial cells was photographed using an inverted microscope. Three independent experiments were performed.

### MTT assay

The antiproliferative effect of γ-tocotrienol against HUVECs and HCC cell lines was determined by the MTT dye uptake method. Briefly, the cells (5 × 10^3^/well) were incubated in triplicate in a 96-well plate in the presence or absence of indicated concentrations of γ-tocotrienol in a final volume of 0.2 ml for 24 h at 37 °C. HUVECs were treated with or without 10 ng/mL VEGF. Thereafter, 20 μl of MTT solution (5 mg/ml in PBS) was added to each well. After a 4 h incubation in the dark at 37°C, 0.1 ml of lysis buffer (20% SDS, 50% dimethylformamide) was added and incubated for 2 h at 37°C, followed by measurement of optical density at 570 nm by Tecan plate reader (Durham, NC).

### Rat aortic ring assay

Rat aortic ring assay was performed as described previously [46]. In brief, aortas isolated from Sprague-Dawley rats were cleaned of fibroadipose tissue and colateral vessels and cut into approximately 1 mm long rings. The aortic rings were randomized into Growth Factor Reduced Matrigel-coated wells and further sealed with a 100 μl overlay of Matrigel. Medium 200 containing with and without VEGF along with different dilutions of γ-tocotrienol was added to the wells and incubated at 37°C/5% CO_2_ for 6 days. At the end of incubation, the microvessel sprouting formed were fixed and photographed using a Nikon inverted microscope (magnification, 100X). Two independent experiments were performed.

### Chick embryo chorioallantoic membrane (CAM) assay

Fertilized chicken eggs were purchased from SSS Exports International (Tamil Nadu, India). According to a previously described method [47], fertilized eggs are incubated at 37.5°C with the relative humidity 60-62% for 72 h. The egg is held horizontally and cracked on the edge, keeping close to the bottom of a petri dish and content of the egg transferred to the petri dish with the embryo and the yolk vessels lying on top of undamaged yolk. As a carrier, a 3x3 mm Whatman filter disk pretreated by 20 μg/disk γ-tocotrienol with or without 100 ng VEGF was put onto the CAM. The ex ovo cultures are returned to an incubator and kept at 38.2°C and 60% humidity for another 5 days. Then the CAM was observed under microscope, and the neovascularization was quantified. Two independent experiments were performed (n=10/group).

### Matrigel plug assay

Matrigel plug assay was performed as described previously [48]. Matrigel (0.5 mL) containing 100 ng VEGF and 20 units of heparin with or without 10 or 20 μg of γ-tocotrienol were injected subcutaneously into the ventral area of C57/BL/6 mice (n=5 per group). After 6 days, the mice were sacrificed using CO_2_ and intact Matrigel plugs from all groups of mice with different treatments were removed. The removed matigel plugs were photographed and then fixed with 10 & neutral buffered formalin and paraffin sections were used for H&E staining to identify the formation and infiltration of new microvessels. Functional microvessels with RBC were quantified manually using a microscope (magnification, 200X).

### Tumor-induced angiogenesis

All procedures were reviewed and approved by the SingHealth IRB and Animal Use and Care Committee. Single cell suspensions freshly derived from HCC patients' xenografts, designated as HCC180811 and HCC020113, were mixed with phenol red-free matrigel and injected into both flanks of severely combined immunodeficient (SCID) mice. For γ-tocotrienol treated group, matrigel was mixed with cells in the presence of 20 μg γ-tocotrienol and 20 units of heparin. Matrigel mixed with the cell suspensions alone was used as negative control. Seven days post-implantation, matrigel plugs were removed and photographed.

### Western blotting

For detection of phopho-proteins, γ-tocotrienol treated cells were harvested and lysed in whole cell lysis buffer (20 mM Tris (pH 7.4), 250 mM NaCl, 2 mM EDTA (pH 8.0), 0.1% Triton X-100, 0.01 mg/ml aprotinin, 0.005 mg/ml leupeptin, 0.4 mM PMSF, and 4 mM NaVO4). Lysates were then spun at 14,000 rpm for 10 min to remove insoluble material and stored at −80°C for later use. The protein content in the lysates was measured by Bio-Rad Protein Assay Dye Reagent Concentrate (Bio-Rad, CA) and equal quantity of protein was resolved on a 10% SDS gel. After electrophoresis, the proteins were electrotransferred to a nitrocellulose membrane, blocked with 5% nonfat milk, and probed with appropriate antibody overnight at 4°C. The blot was washed, exposed to HRP-conjugated secondary antibodies for 1 h, and finally examined by chemiluminescence (ECL; GE Healthcare, Buckinghamshire, UK).

### Orthotopic HCC mouse model

All procedures involving animals were reviewed and approved by the SingHealth Animal Use and Care Committee. Ten week-old Balb/c nude female mice (Biolasco, Taiwan) were implanted orthotopically with approximately 1 mm^3^ of human HCCLM3_Luc2 tumor stably expressing firefly luciferase. Tumor growth was monitored bi-weekly by bioluminescence imaging using IVIS 200 Bioluminescence Imaging System (Xenogen Corp., Alameda, CA). Mice were randomized and treatment was started once the observed bioluminescent signal increased steadily. Mice were administered 3.25 mg γ-tocotrienol in the form of self-emulsifying mixture by oral gavage 5 days a week. For control, γ-tocotrienol in the formulation was substituted by corn oil. All mice were euthanized once the tumor signal reached above 1 × 10^11^ photons per second (p/s), and tumor were harvested for subsequent analysis. For imaging, mice were given i.p. injections of 150 mg/kg D-luciferin (Xenogen) 10 minutes before imaging. To quantitate tumor burden, bioluminescence signals were calculated from the imaging data using the Living Image software 3.2 (Xenogen) according to manufacturer's protocol.

### Immunohistochemical analysis of tumor samples

Solid tumors from control and treated mice were fixed with 10% phosphate buffered formalin, processed and embedded in paraffin. Sections were cut and deparafinized in xylene, and dehydrated in graded alcohol and finally hydrated in water. Antigen retrieval was performed by boiling the slide in 10 mM sodium citrate (pH 6.0) for 30 min. Immunohistochemistry was performed following manufacturer instructions (DAKO LSAB kit). Briefly, endogenous peroxidases were quenched with 3% hydrogen peroxide. Non-specific binding was blocked by incubation in the blocking reagent in the LSAB kit (Dako, Carpinteria, CA) according to the manufacturer's instructions. Sections were incubated overnight with primary antibodies as follows: Ki-67, VEGF, CD31, and anti-cleaved caspase-3 (each at 1:100 dilution). Slides were subsequently washed several times in Tris buffered saline with 0.1% Tween 20 and were incubated with biotinylated linker for 30 min, followed by incubation with streptavidin conjugate provided in LSAB kit (Dako) according to the manufacturer's instructions. Immunoreactive species were detected using 3, 3-diaminobenzidine tetrahydrochloride (DAB) as a substrate. Sections were counterstained with Gill's hematoxylin and mounted under glass cover slips. Images were taken using an Olympus BX51 microscope (magnification, 40X).

### Statistical analysis

Data are expressed as the mean ± S.D. In all figures, vertical error bars denote the S.D. The significance of differences between groups was evaluated by Student's t-test and one way analysis of variance, (ANOVA) test. A p value of less than 0.05 was considered statistically significant.

### Abbreviations

HCC, hepatocellular carcinoma; HUVEC, human umbilical vein endothelial cells; VEGF, vascular endothelial growth factor; VEGFR2, vascular endothelial growth factor receptor 2; mTOR, mammalian target of rapamycin; NF-κB, nuclear factor-kappa B; STAT3, signal transducer and activator of transcription 3; CAM, chick chorioallantoic membrane; MTT, 3-(4,5-dimethylthiazol-2-yl)-2,5-diphenyl-2H-tetrazolium bromide; PARP, Poly (ADP-ribose) polymerase; SCID, severe combined immunodeficiency; HIF, hypoxia-inducible factor; PTEN, phosphatase and tensin homologue.
